# Environmental Investigations and Tissue Bioaccumulation of Heavy Metals in Grey Mullet from the Black Sea (Bulgaria) and the Ionian Sea (Italy)

**DOI:** 10.3390/ani10101739

**Published:** 2020-09-24

**Authors:** Francesco Fazio, Claudio D’Iglio, Gioele Capillo, Concetta Saoca, Katya Peycheva, Giuseppe Piccione, Lubomir Makedonski

**Affiliations:** 1 Department of Veterinary Sciences, Polo Universitario Annunziata, University of Messina, 98168 Messina, Italy; gcapillo@unime.it (G.C.); csaoca@unime.it (C.S.); gpiccione@unime.it (G.P.); 2 Institute for Marine Biological Resources and Biotechnology (IRBIM), National Research Council (CNR), Section of Messina, Spianata S. Raineri 86, 98122 Messina, Italy; claudio.diglio@irbim.cnr.it; 3 Department of Chemistry, Faculty of Pharmacy, Medical University Varna, 84 Tzar Osvoboditel Blv, 9000 Varna, Bulgaria; peytcheva@hotmail.com (K.P.); lubomir60@yahoo.com (L.M.)

**Keywords:** environmental investigation, Black Sea, Ionian Sea, *Mugil cephalus*, heavy metals

## Abstract

**Simple Summary:**

The environmental monitoring of dangerous chemicals and how these affect the aquatic biota is of fundamental importance in defining the health status of fish. Pollution with chemical elements is of great environmental concern, since fish and marine organisms can uptake various toxicants and subsequently transfer them to man through the food web. Moreover, the accumulation of toxic elements could be a cause of pathology insurgence in fish. These organisms represent a good indicator of the status of coastal water. Flathead grey mullet (*Mugil cephalus*) is a coastal species, bottom dwelling and feeding on detritus, invertebrates, and algae. The main aim of the present study was to determine the total concentration of nine elements (Cd, Cr, Pb, Ni, Al, Cu, Fe, Mn, and Zn) in the fish species *M. cephalus* and in coastal marine waters collected from various sampling points along the Black Sea (Bulgaria) and the Ionian Sea (Italy) and to apply those results to the prediction of the pollution status of those coastal marine environments. To achieve this goal, metal concentrations were analyzed in various fish tissues (gills, liver, and muscle) of grey mullet (*M. cephalus*) and in marine water samples collected from the sampling stations across both areas (Ionian Sea (Italy) and Black Sea (Bulgaria)). The results revealed significant differences within the tissues examined and the marine water samples, principally attributable to the pollution of the area, the bioavailability of metals, and the hydrological conditions. The present study represents the first attempt to compare the data obtained from analyzing sampling points in order to define the different elemental concentrations in *M. cephalus* muscle tissue and how they reflect environmental ones.

**Abstract:**

The environmental monitoring of chemical toxicants has been a widely studied topic in the last few decades. The main aim of the present study was to determine the total concentration of nine elements (Cd, Cr, Pb, Ni, Al, Cu, Fe, Mn, and Zn) in the fish species grey mullet (*M. cephalus)* and in the coastal marine waters collected from various sampling points along the Black Sea (Bulgaria) and the Ionian Sea (Italy). Further, those results were applied to predict the pollution degree in those coastal marine environments. The fish samples were subject to acid digestion followed by appropriate analytical determination. The metal concentrations in marine water samples collected from the Black Sea (Bulgaria) and the Ionian Sea (Italy) were also analyzed. Unpaired Student’s t-test and the one-way ANOVA were applied for the statistical analysis of the data. The statistical results revealed a significant variation (*p* < 0.0001) in the concentration of various fish tissues. The accumulation of toxic and essential elements differs significantly in grey mullet species caught from the Black Sea (Bulgaria) and the Ionian Sea (Italy). The results from this study may serve as a convenient approach during marine pollution programs set by both countries (Italy and Bulgaria).

## 1. Introduction

Pollution with heavy metals is of great environmental concern, since fish and other aquatic organisms can accumulate various toxicants and subsequently transfer them to man through the food web. These organisms represent a good indicator of the status of coastal water [1,2,3,4,5].

Heavy metal uptake is strictly related to the species considered. It depends on the sex, age, size, reproductive cycle, swimming pattern, feeding behavior, hydrological conditions, and geographical location of the fish species [6,7].

Using various marine organisms such as fish for a biomonitoring purpose has many advantages, since these organisms are able to accumulate only the biologically available forms of the heavy metals dissolved in the aquatic environment. Moreover, fish enable the continuous monitoring of the aquatic medium due to their characteristic of uptaking contaminants, also in cases where trace pollutants are near to the detection limits [2].

Heavy metals can be classified as potentially toxic (arsenic, cadmium, lead, mercury, nickel, etc.), probably essential (vanadium, cobalt), and essential (copper, zinc, iron, manganese, selenium) [8]. Toxic elements can be very harmful even at low concentrations when ingested over a long time, and may affects the life cycle stages of fish [9,10]. Additionally, it has been demonstrated that various toxic elements are linked with skeletal deformities in natural fish populations as well as in laboratory-produced ones [11], alterations in lateral lines [12], and effects on hematological and biochemical parameters [13,14]. Essential metals, on the other hand, can also produce toxic effects when the metal intake is excessively elevated [15,16].

The behavior and ecological characteristics of the grey mullet (*Mugil cephalus*) species makes it suitable for the monitoring of marine coastal metal pollution [17,18,19]. Grey mullet is a bottom-dwelling species that feeds on detritus, microscopic invertebrates, and algae [2,20], and it is known to accumulate a high level of heavy metals in its organs from the surrounding environment [21].

The Black Sea is known to be the largest natural anoxic water basin (below 180 m in depth) in the world. It has very few seawater exchanges with the Mediterranean Sea. However, the Black Sea receives several freshwater inputs from some of Europe’s largest rivers (the Danube, Dniester, and Dnieper) [22]. This, jointly with numerous human activities in the area, has led to it having a highly polluted condition.

The Ionian Sea forms the westernmost part of the eastern Mediterranean basin. The profile of the Ionian Sea has been described by several studies [23,24]. Continuous water exchange and renovation allow a rapid dispersion of contaminants. 

The concentrations of heavy metals in fish have been extensively studied over the past several decades, but still there is a lack of information in the scientific literature concerning the heavy metal distribution in the grey mullet species. The aim of the present study was to determine the total concentration of nine heavy elements (Cd, Cr, Pb, Ni, Al, Cu, Fe, Mn, and Zn) in the fish species *M. cephalus* and in coastal marine waters collected from various sampling points along the Black Sea (Bulgaria) and the Ionian Sea (Italy). Further, we aimed to apply those results to the prediction of the pollution status in those coastal marine environments. To achieve this goal, the metal concentrations were analyzed in various fish tissues (gills, liver, and muscle) of grey mullet (*M. cephalus*) and in marine water samples collected from the sampling stations across both areas (Ionian Sea (Italy) and Black Sea (Bulgaria)).

## 2. Materials and Methods 

### 2.1. Fish Sampling

**Figure 1:**
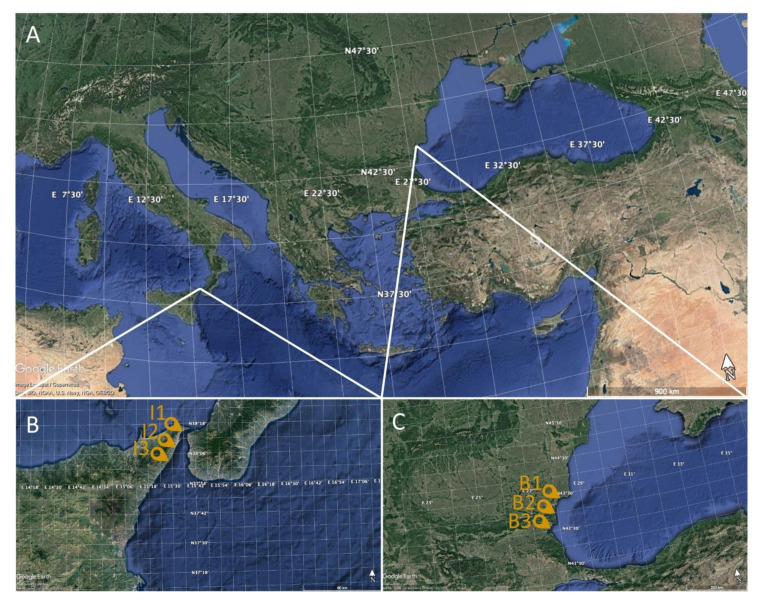
Maps indicating different sampling points. (**A**) Central-eastern Mediterranean Sea. (**B**) Ionian Sea (Italy) (sampling stations I1, I2, T3), (**C**) Black Sea (Bulgaria) (sampling stations B1, B2, B3).

The specimens were caught by bottom-set nets during April 2019. The sampling stations are shown in Figure 1. The sampling stations were selected based on their degree of pollution. The three sampling points across the Ionian Sea were located in an area falling into the Messina Strait boundaries. This area is widely described in the literature [23,25,26,27,28], and it is characterized by strong tidal and stationary currents that continuously mix the Tyrrhenian and Ionian seawater. The sampling points across the Bulgarian Black Sea are recognized as highly populated and industrialized cities.

After capturing, the fish were euthanized using the procedure reported by Iaria et al. [29], modified using 2-phenoxyethanol, and followed by animal welfare procedures with respect to the 2010/63 EU directive [30]. Then, the fish were brought to the laboratory using an icebox, measured with an ichthiometer, and weighted by an electronic balance FX-3000 (max 3100 g precision d = 0.01 g, A & D, 1756 Automation Parkway, San Jose, CA, USA). All the fish were considered healthy based on an external examination for any signs of abnormalities or parasite infestations.

### 2.2. Sample Preparation, Tissue and Water Analysis

The total lengths and weights of the grey mullet samples brought to the laboratory were measured prior to analysis. The muscles, gills, and liver were dissected and included in representative samples. The samples were weighted, packed in polyethylene bags, and stored at −18 °C until chemical analysis. The biometrics data of the analyzed fish included in this study are shown in Table 1.

Before analysis, the samples were thawed at room temperature and each one was dissected using a stainless steel knife. Each specimen (approximately a portion of 5 g of tissues of interest (muscle, gills, and liver)) was collected and weighed in an analytical balance (precision of 0.1 mg).

The samples of tissue were then subjected to wet digestion, performed in triplicate. Approximately 1.0 g portions of each fish tissue (muscle, liver, and gills) were digested by adding 10 mL of HNO3 (65% Merck, Suprapur, Darmstadt, Germany) in a microwave digestion system MARS 6 (CEM Corporation, Matthews, NC, USA), delivering a maximum power and temperature of 800 W and 200 °C, respectively, and internal temperature control was used to assist the acid digestion process. After complete digestion, the samples were diluted to 25 ml with ultrapure water (resistivity 18 M MΩ; Millipore, Bedford, MA, USA) and stored in pre-cleaned polyethylene bottles until the analysis of the metal concentrations. The blanks were prepared in the same way and were used to calibrate the instrument.

The concentrations of Al, Cd, Cr, Cu, Fe, Mn, Ni, Pb, and Zn in the samples were determined using an ICP-OES Spectrometer (Optima 8000, Perkin Elmer, Waltham, MA, USA) with a plasma gas flow of −10 L/min, an auxiliary gas flow of −0.7 L/min, a nebulizer gas flow of −0.2 L/min, and an axial plasma view. The accuracy of the applied analytical procedure for the determination of heavy metals in fish was tested using DORM-4 (NIST)-certified reference material. Recoveries of between 90.5% and 108% were accepted to validate the calibration.

The marine water samples were collected using polyethylene terephthalate bottles. The collection depth was about 3 m from the surface. The samples were then transferred to the laboratory using a lightproof insulated box containing ice packs.

The heavy metal determination in marine water samples was carried out using atomic absorption spectroscopy (AAS) (Perkin Elmer/AAnalyst 400, Waltham, MA, USA). Different concentrations of standard solutions were carried out to calibrate the AAS for all the metals.

### 2.3. Statistical Analysis

Prior to statistical analysis, all the data were tested for normal distribution with the Kolmogorov–Smirnov test. All the data were normally distributed and a statistical analysis was performed on the mean values.

AN unpaired Student’s *t*-test was used to determine significant differences between the metal concentrations (Cd, Cr, Pb, Ni, Al, Cu, Fe, Mn, and Zn) in water samples from the Black Sea versus the Ionian Sea and to compare the metal contents in the water of the two sites. 

A one-way analysis of variance (ANOVA) was applied to find significant differences in the metal concentrations of the tissues in grey mullet caught from the Black Sea (Bulgaria) and the Ionian Sea (Italy). Additionally, the ANOVA test was used to assess the differences among metal accumulation in different tissues of grey mullet from the same environment. The level of significance was set at *p* < 0.05. All the statistical calculations were performed with the statistical software program Prism v. 7.00, 2003 (Graphpad Software Ltd., San Diego, CA, USA).

## 3. Results

Table 2 shown the heavy metal concentrations (mean ± SD) measured in the seawater of the Black Sea (Bulgaria) and the Ionian Sea (Italy). 

The distribution of the different heavy metals along both sampling sites varied considerably, with no clear pattern detected. The most abundant heavy metal in the water samples from the Black Sea was zinc (22.18 μg/L), while that in the Ionian Sea was copper (41.27 μg/L).

**Figure 2:**
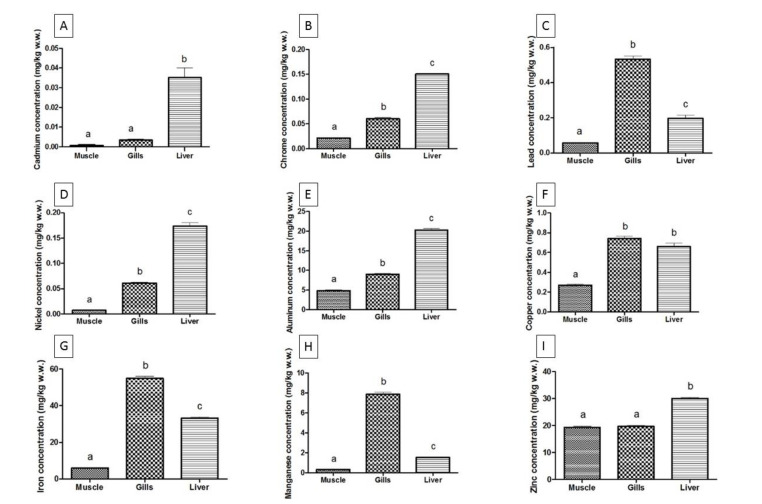
Metal concentration in different tissues of *Mugil cephalus* (Linnaeus, 1758) from the Ionian Sea, Italy. Different alphabetical characters represent statistical differences (*p* < 0.05). From (**A**–**I**) are plotted metal concentrations in the following order: Cd, Cr, Pb, Ni, Al, Cu, Fe, Mn, and Zn.

**Figure 3:**
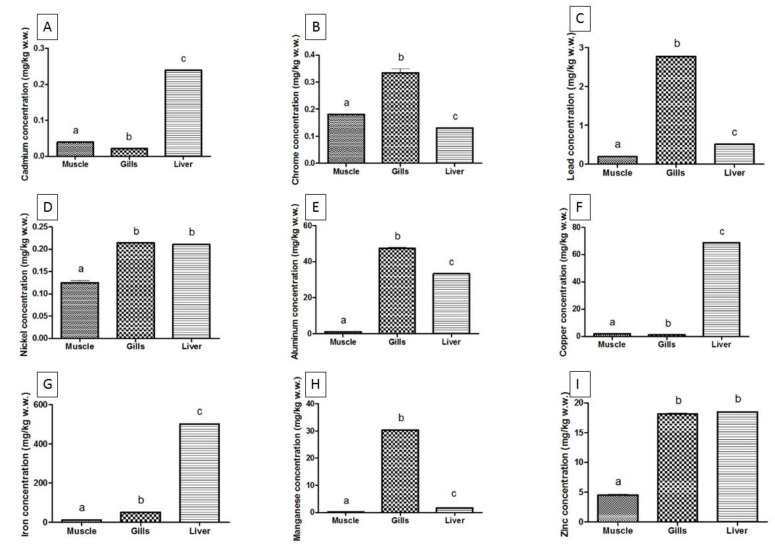
Metal concentrations in different tissues of *Mugil cephalus* (Linnaeus, 1758) from the Black Sea, Bulgaria. Different alphabetical characters represent statistical differences (*p* < 0.05). From (**A**–**I**) are plotted metal concentrations in the following order: Cd, Cr, Pb, Ni, Al, Cu, Fe, Mn, and Zn.

Table 3 shows the heavy metal concentrations (mean ± SD) in different *M. cephalus* tissues for each sampling site in the Black Sea and the Ionian Sea. The heavy metal levels of the grey mullet from the Black Sea (Bulgaria) for all the tissues studied have the following pattern: Fe > Al > Cu > Zn > Mn > Pb > Cr > Ni > Cd. Meanwhile, those from the Ionian Sea (Italy) have the pattern: Fe > Zn> Al > Mn > Cu > Pb > Ni > Cr > Cd. The accumulation patterns of all metals were significantly different (*p* < 0.001) between the different species, organs, and sites (except for Fe), as well as the different sampling sites (Figure 2 and Figure 3).

All the heavy metals studied (except Zn) exhibited higher concentrations in the analyzed tissues of mullet caught in the Black Sea, Bulgaria, compared to those from the Ionian Sea, Italy.

Additionally, the Black Sea mullet’s liver showed the following maximum concentrations: Cd (0.25 mg/kg *w*.*w*), Cu (69.30 mg/kg *w*.*w*), Fe (505.75 mg/kg *w*.*w*). The gills of the same species caught from the Black Sea had high levels of Cr (0.49 mg/kg *w*.*w*), Pb (2.91 mg/kg *w*.*w*), Al (52.04 mg/kg *w*.*w*), and Mn (31.52 mg/kg *w*.*w*). On the contrast, the liver tissues of the grey mullet from the Ionian Sea showed high levels of Ni (0.25 mg/kg *w*.*w*) and Zn (34.00 mg/kg *w*.*w*). The different levels of heavy metals in the different tissues of the grey mullet is due to the different pattern of tissue accumulation, as shown by various authors [31,32].

In Figure 2, the data of the heavy metals determined and their relative statistical significance for the mullet caught from the Ionian Sea are plotted. The accumulation of heavy metals was predominantly in the liver tissue of *M. cephalus* for both the Black and Ionian seas. Significant statistical differences resulted for the most of the relations between the heavy metal concentrations in different tissues, except for Cd in the muscle and gills (Figure 2A), Cu in the gills and liver (Figure 2F), and Zn in the muscle and gills (Figure 2I).

Figure 3 shows the graphical elaborations of the heavy metal concentrations in the studied tissues from *M. cephalus* collected in the Black Sea (Bulgaria). In a few cases, the statistical differences were not significant between the metal concentrations in different tissues, including Ni and Zn in the gills and liver.

## 4. Discussion

The concentrations of heavy metals detected in the fish of this study were compared with the other reported values in an effort to determine the degree of contamination in the study areas. The reported results in the literature showed that the metal contents in the fish muscles varied widely depending on where the specimens were caught.

As is shown in Figure 2 and Figure 3, the concentration of heavy metals in the muscle tissue is generally lower than that in the gills and liver. These results are in agreement with the ones reported by other authors [33,34,35,36,37]. Dural et al. [38] reported that Cd, Pb, Cu, Zn, and Fe were found with the highest level in muscle tissue in *S. aurata*, while *D. labrax* and *M. cephalus* accumulated the lowest amounts of heavy metals in the muscle. 

Heavy metals are absorbed, stored, and detoxified in excess by the fish in the liver [39,40]. Liver is known to be the target organ for the accumulation and detoxification of most metals, independently of the uptake route, representing the optimal tissue for water monitoring. The liver is the organ that maintains for a long time higher concentrations of metals, reflecting proportionally the environmental ones [41].

The gills represent the organ where gas exchange takes place and are involved in the excretion of metal ions, which are excreted in the surrounding water from the body. Furthermore, the mucous excretion of gills has a high binding affinity with metal ions [42]; this and the high metal storage capacity of the gills during their function of absorption and depuration [43,44,45] justified the high concentration of heavy metals found in this tissue.

The higher concentrations of analyzed metals observed in the gills and liver with respect to the muscle are also due to the ability of metallothioneins (proteins synthesized in these organs when fish are exposed to heavy metals) to bind metals and facilitate their detoxification, protecting fish from heavy metal damage [37,45]. The higher heavy metal level in the gills compared to the liver may indicate temporary peaks of the heavy metal concentration in the surrounding waters. Additionally, the high levels of some heavy metals in the liver are related to cases with chronic or long-term heavy metal pollution. Therefore, the low concentrations of metals observed in the muscles would be due to the low levels of binding proteins in these tissues [46].

Moreover, it is widely recognized that muscle tissue does not accumulate most metals, except for mercury, which has a good affinity for this tissue [41,47].

Regarding the metal concentrations in seawater, the present study revealed that significant variations in the metal levels existed in the water of the two sampling regions studied, with a higher concentration of toxic and essential elements in the Ionian Sea with respect to the Black Sea. Despite the fact that the water analyses revealed a higher concentration of most of the studied metals in the Ionian Sea with respect to the Black Sea, this was not confirmed by the grey mullet tissue concentrations. A good explanation of this is that the bioconcentration of contaminants depends on water circulation and renewal. The geographical and hydrological characteristics of the studied environments affects the bioconcentration of such a contaminant. The Black Sea is, in fact, an almost closed basin, with numerous freshwater inputs coming from high-dimension and high-polluted rivers rather than smaller sources in all the Black Sea coastal countries [48]. Once they reach the sea, contaminants in the Black Sea tend to settle in the coastal water, especially because of the scarce water circulation, entering then into food webs, becoming bioavailable, and accumulating in fish species [49]. In contrast, the contaminants in open seas such as the Ionian Sea are continuously dispersed by water circulation and currents. The sampling area of the Ionian Sea is influenced by the high hydrodynamics of Messina’s Strait. Tidal and stationary currents daily renew the coastal water of Messina’s Strait, allowing the dispersion of dissolved metals [26,50,51,52,53]. In addition, the lower concentration of metals in the grey mullet tissues from the Ionian Sea can be due to the low bioavailability of metals in Ionian seawater. It is in fact clear that the bioavailable fractions of metals infer the uptake rates of these contaminants [49]. Pan and Wang [54] demonstrated that direct measurements of metal concentrations in marine environments and food phases cannot provide precise information about metal bioavailability. 

The non-significant differences in metals found in seawater from different sampling points in the same area confirm the uniformity of coastal water composition in terms of metal concentrations. 

The present study reveals higher concentrations of all the heavy elements studied (except for Zn) for the mullets caught from the Black Sea (Bulgaria) compared to those from the Ionian Sea (Italy).

Several authors have previously investigated the feeding habits and heavy metal distributions in grey mullet (*M. cephalus*), since this fish species lives close to the bottom sediments [55,56,57,58,59]. They found that the mullet specimens accumulate heavy metals in various tissues, and this is a prominent indicator for the heavy metal pollution coming from the surrounding water bodies. 

The levels of metals in the tissues of grey mullet collected in different regions may indicate the current pollution status concerning the metals of the area where the fish are caught. Our results provide useful information on the distribution of metals in the muscle, gills, and liver of grey mullet caught in the Black Sea and the Ionian Sea. They indicated that metals have a different degree of accumulation in different tissues of this species and in specimens coming from different localities, confirming that geographical locations can determine different metal concentrations in the same fish species, as previously documented by several authors [2,37].

## 5. Conclusions

The present study provides novel information about the distribution of heavy metals in *M. cephalus* and marine water collected from two different European regions—the Black Sea (Bulgaria) and the Ionian Sea (Italy). The results from the study show that the different accumulation levels of the analyzed metals in various tissues of *M. cephalus* caught from the Black Sea (Bulgaria) and the Ionian Sea (Italy) might be used as good tool for coastal water monitoring, independently of the coastal area.

Moreover, the present paper attempts to define a baseline dataset for *M. cephalus* muscle tissue metal concentrations for both environmental and fish health.

Additionally, the results from this study may serve as a convenient approach during the marine environmental monitoring and pollution programs set by both countries (Italy and Bulgaria). 

## Figures and Tables

**Table 1:** Biometrics data (mean ± SD) of the grey mullet sampled from the coastal waters of the Black Sea (Bulgaria) and the Ionian Sea (Italy).

Sample	Sampling Site	N	Sampling Stations	Weight (g) ± SD	Length (cm) ± SD
Gray mullet (*Mugil cephalus*)	Black Sea (Bulgaria)	20	B1, B2, B3	104.33 ± 13.80	23.17 ± 2.61
	Ionian Sea (Italy)	20	I1, I2, I3	97.12 ± 11.25	22.41 ± 2.38

SD: Standard Deviation.

**Table 2:** Trace element concentration (mean ± SD) measured in the seawater of the Black Sea (Bulgaria) and the Ionian Sea (Italy).

Trace Elements (μg/L)	Sampling Sites	
	Black Sea (Bulgaria)	Ionian Sea (Italy)
Cd	0.0445 ± 0.0020	0.9289 ± 0.0095 *
Cr	0.3751 ± 0.0044	0.9042 ± 0.0148 *
Pb	0.3472 ± 0.0006	0.8060 ± 0.1095 *
Ni	0.5286 ± 0.0113	0.8435 ± 0.0440 *
Al	5.4634 ± 0.2208	0.9350 ± 0.0215 *
Cu	0.5340 ± 0.0080	41.2700 ± 0.2720 *
Fe	3.4591 ± 0.1834	0.8920 ± 0.0507 *
Mn	0.5388 ± 0.0307	0.9385 ± 0.0048 *
Zn	22.1829 ± 0.1049	0.8821 ± 0.0379 *

* Shows significance (*p* < 0.0001); SD: Standard Deviation.

**Table 3:** Trace element concentrations (mean ± SD) of the various tissues measured in the grey mullet *Mugil cephalus* (Linnaeus, 1758) caught from the Ionian Sea (Italy) and the Black Sea (Bulgaria).

Heavy metals (μg/L)	Samples	Muscle	Gills	Liver
Cd	Ionian Sea	0.0008 ± 0.0010 ^a^	0.0034 ± 0.0021 ^a^	0.0351 ± 0.0217 ^a^
	Black Sea	0.0391 ± 0.0043 ^b^	0.0214 ± 0.0058 ^b^	0.2391 ± 0.0034 ^b^
Cr	Ionian Sea	0.0213 ± 0.0012 ^a^	0.0602 ± 0.0106 ^a^	0.1506± 0.0066 ^a^
	Black Sea	0.1809 ± 0.0051 ^b^	0.3340 ± 0.0678 ^b^	0.1303 ± 0.0027 ^b^
Pb	Ionian Sea	0.0572 ± 0.0104 ^a^	0.5320 ± 0.0844 ^a^	0.1970 ± 0.0771 ^a^
	Black Sea	0.1914 ± 0.0046 ^b^	2.768 ± 0.0525 ^b^	0.5118 ± 0.0098 ^b^
Ni	Ionian Sea	0.0071 ± 0.0011 ^a^	0.0610 ± 0.0079 ^a^	0.1735± 0.0315 ^a^
	Black Sea	0.1249 ± 0.0211 ^b^	0.2144 ± 0.0033 _b_	0.2111 ± 0.0062 ^b^
Al	Ionian Sea	4.8500 ± 0.8082 ^a^	9.0230 ± 0.7341 ^a^	20.2900 ± 1.9230 ^a^
	Black Sea	1.0210 ± 0.0061 ^b^	47.3800 ± 2.5700 ^b^	33.3100 ± 0.4651 ^b^
Cu	Ionian Sea	0.2695 ± 0.0473 ^a^	0.7400 ± 0.1114 ^a^	0.6600 ± 0.1635 ^a^
	Black Sea	1.8270 ± 0.0151 ^b^	1.1760 ± 0.0385 ^b^	68.6200 ± 0.3200 ^b^
Fe	Ionian Sea	5.9250 ± 0.9792 ^a^	54.9100 ± 5.1440 ^a^	33.1500 ± 3.2970 ^a^
	Black Sea	11.0600 ± 1.6310 ^b^	50.8900 ± 1.6730 ^b^	502.3000 ± 2.0410 ^b^
Mn	Ionian Sea	0.3040 ± 0.0484 ^a^	7.8550 ± 0.9310 ^a^	1.5350 ± 0.2455 ^a^
	Black Sea	0.1376 ± 0.0183 ^b^	30.3300 ± 0.7803 ^b^	1.6710 ± 0.0204 ^b^
Zn	Ionian Sea	19.3000 ± 2.0800 ^a^	19.6000 ± 1.7890 ^a^	30.0000 ± 1.8920 ^a^
	Black Sea	4.5330 ± 0.5551 ^b^	18.1100 ± 0.7027 ^b^	18.4800 ± 0.4849 ^b^

Means without the same alphabetical characters (a, b) within the same parameter and tissue represent statistical differences (*p* < 0.0001).
